# Wnt/β-catenin signaling pathway induces autophagy-mediated temozolomide-resistance in human glioblastoma

**DOI:** 10.1038/s41419-020-02988-8

**Published:** 2020-09-17

**Authors:** Eun-Jin Yun, Sangwoo Kim, Jer-Tsong Hsieh, Seung Tae Baek

**Affiliations:** 1 POSTECH Biotech Center, POSTECH, Pohang, Republic of Korea; 2 Department of Biomedical Systems Informatics and Brain Korea 21 PLUS Project for Medical Science, Yonsei University College of Medicine, Seoul, Republic of Korea; 3 Department of Urology, University of Texas Southwestern Medical Center, Dallas, TX USA; 4 Department of Biotechnology, Kaohsiung Medical University, Kaohsiung, Taiwan Republic of China; 5 Institute for Convergence Research and Education in Advanced Technology, Yonsei University, Seoul, Republic of Korea; 6 Department of Life Sciences, POSTECH, Pohang, Republic of Korea

**Keywords:** CNS cancer, Molecular biology

## Abstract

Temozolomide (TMZ) is widely used for treating glioblastoma multiforme (GBM), however, the treatment of such brain tumors remains a challenge due to the development of resistance. Increasing studies have found that TMZ treatment could induce autophagy that may link to therapeutic resistance in GBM, but, the precise mechanisms are not fully understood. Understanding the molecular mechanisms underlying the response of GBM to chemotherapy is paramount for developing improved cancer therapeutics. In this study, we demonstrated that the loss of DOC-2/DAB2 interacting protein (DAB2IP) is responsible for TMZ-resistance in GBM through ATG9B. DAB2IP sensitized GBM to TMZ and suppressed TMZ-induced autophagy by negatively regulating ATG9B expression. A higher level of ATG9B expression was associated with GBM compared to low-grade glioma. The knockdown of ATG9B expression in GBM cells suppressed TMZ-induced autophagy as well as TMZ-resistance. Furthermore, we showed that DAB2IP negatively regulated ATG9B expression by blocking the Wnt/β-catenin pathway. To enhance the benefit of TMZ and avoid therapeutic resistance, effective combination strategies were tested using a small molecule inhibitor blocking the Wnt/β-catenin pathway in addition to TMZ. The combination treatment synergistically enhanced the efficacy of TMZ in GBM cells. In conclusion, the present study identified the mechanisms of TMZ-resistance of GBM mediated by DAB2IP and ATG9B which provides insight into a potential strategy to overcome TMZ chemo-resistance.

## Introduction

Glioblastoma multiforme (GBM) is the most prevalent primary malignant brain tumor and, remains one of the most lethal cancers, with a median survival of only 15 months upon diagnosis^1^. The standard care for GBM includes maximally safe surgical resection followed by radiation therapy and concurrent temozolomide (TMZ) chemotherapy^2^. TMZ is a DNA alkylating agent known to induce several DNA adducts by methylating DNA at different positions, which can interfere with DNA replication and induce cell cycle arrest at G2/M, consequently leading to apoptosis^3,4^. Concomitant therapy using TMZ and radiation has improved the prognosis for GBM patients. However, the average survival expectancy remains 14.6 months, including 2.5 months of increased median survival^2,5^. GBMs eventually relapse after treatments, moreover, not all patients respond to this therapy mainly due to TMZ-resistance, and the chemo-resistance remains one of the major clinical challenges^2,5^. Therefore, understanding the molecular mechanisms contributing to this resistant phenotype will provide important insight for developing novel therapeutic approaches and identifying those patients most likely to benefit from a specific treatment regimen. The overexpression of the demethylating enzyme *O*^6^-methylguanine-DNA methyltransferase (MGMT), removing alkyl groups from the *O*^6^ position of guanine directly, has been implicated as one mechanism of TMZ-resistance^6^. While MGMT remains a major cause of TMZ-resistance, some MGMT-deficient GBMs are still resistant to TMZ suggesting that other components may also be involved in TMZ-resistance. However, the underlying molecular mechanisms remain unclear^3,4,6^.

Meanwhile, the DOC-2/DAB2 interacting protein DAB2IP was previously identified as a member of the Ras-GTPase activating protein family, and functions as a tumor suppressor in cancer progression^7,8^. In various cancer types, DAB2IP has been shown to control cell proliferation, apoptosis, and cell survival by inhibiting the Ras/MAPK pathway, activating the Ask1/JNK pathway, and inhibiting the PI3K/Akt pathway, respectively^7,9,10^. As a Ras-GAP, DAB2IP is highly expressed in developing and adult brains, where it has been shown to interact with Reelin transducing protein Dab2, and function in regulating synaptogenesis, dendritic morphogenesis, and neuronal differentiation and migration^8,11–13^. However, the physiological and pathological function of DAB2IP in GBM is relatively unknown. Previously, we demonstrated that the loss of DAB2IP expression resulted in increased chemo-resistance as well as radioresistance in some cancer types^14–16^. Interestingly, we observed that the loss of DAB2IP in GBM cells increased their chemo-resistance to TMZ.

Here, we demonstrated a link between the loss of DAB2IP and TMZ-resistance. Mechanistically, DAB2IP sensitized GBM to TMZ and suppressed TMZ-induced autophagy by negatively regulating ATG9B via the Wnt/β-catenin pathway. Combination treatment of GBM cells using a small molecule inhibitor blocking the Wnt/β-catenin pathway in addition to TMZ synergistically enhanced the efficacy of TMZ. Our results showed that DAB2IP could function as a chemo-sensitizer to TMZ in combination treatment by suppressing Wnt/β-catenin-mediated autophagy.

Overall, these findings demonstrate a link between the loss of DAB2IP and TMZ-resistance mechanisms and offer a potential therapeutic strategy to re-sensitize chemo-resistant GBM.

## Results

### DAB2IP is downregulated in TMZ-resistant glioblastoma cells

**Fig. 1:**
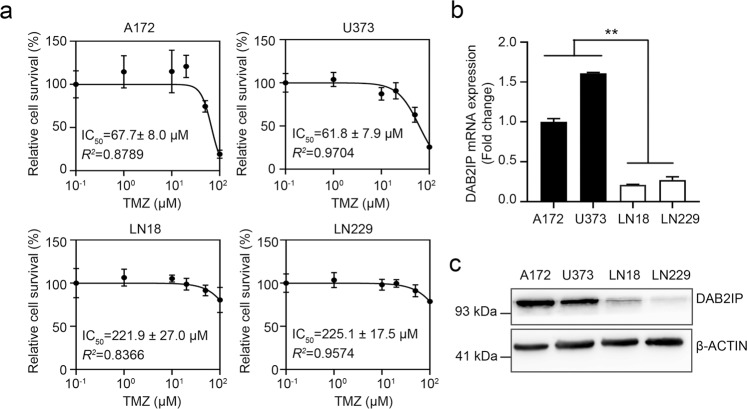
TMZ-resistant GBM cell lines express a low level of DAB2IP. **a** TMZ log–dose–response analysis (IC_50_) in four human glioblastoma cell lines. Cells were treated with increment doses of TMZ as indicated for 48h, and IC_50_ was determined with cytotoxicity results assessed by MTT assay. Percent survival curves after TMZ treatment was constructed by plotting the cell survival whose values indicate mean ± SD (*n* = 5). Error bars indicate standard deviation. **b** The mRNA expression of DAB2IP analyzed by real-time PCR in four human glioblastoma cell lines. Black and white bars indicate DAB2IP-high and DAB2IP-low cell lines, respectively. Means ± SD; *n* = 3; Student’s two-tailed *t*-test, **p < 0.01. **c** Representative images of western blot against DAB2IP and β-actin.

The IC_50_ values for TMZ in GBM cell lines vary in many studies depending on the experimental conditions^4^. Thus we determined the responsiveness of GBM cells to TMZ in our system. As shown in Fig. 1a, the IC_50_ for TMZ ranged from 61.8 ± 7.9 to 225.1 ± 17.5 μM and fell into two groups, the TMZ-sensitive (A172 and U373; <100 μM) and the TMZ-resistant groups (LN18 and LN229; >200 μM). We then compared the expression pattern of DAB2IP in the TMZ-sensitive and TMZ-resistant cells. DAB2IP, a novel tumor suppressor, is often downregulated in advanced stages of many types of cancer, and the loss of DAB2IP expression has been shown to correlate with the poor prognosis and increased tumor metastasis of many malignancies^7,8^. In addition, according to previous studies, DAB2IP has critical roles in suppressing chemo-resistance and radioresistance in different types of cancer^14–16^. To investigate whether DAB2IP is involved in TMZ sensitivity in GBM as well, we screened the expression level of DAB2IP in GBM cell lines that respond differently to TMZ. We observed TMZ-resistant cells expressed a low level of DAB2IP in both RNA and protein levels (Fig. 1b, c) indicating a possible link between the level of DAB2IP and chemo-resistance to TMZ in GBM cell lines.

### Downregulation of DAB2IP contributes to TMZ-resistance

**Fig. 2:**
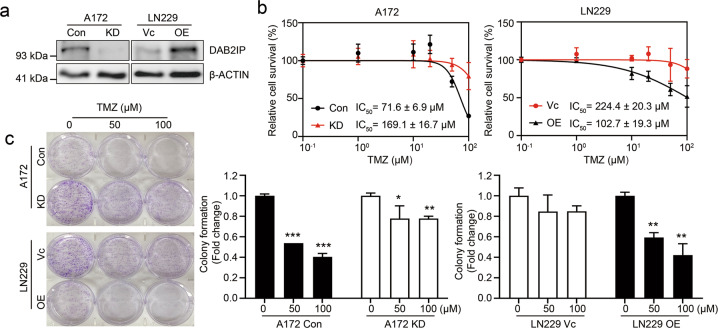
DAB2IP sensitizes GBM cells against to TMZ. **a** Representative images of western blot against DAB2IP and β-actin. A172 cell was transfected with pGIPZ-shControl (Con) or pGIPZ-shDAB2IP (KD) to knockdown DAB2IP, and LN229 cell was transfected with pcDNA3.1-vector (Vc) or pcDNA3.1-DAB2IP (OE) to overexpress DAB2IP. **b** TMZ log–dose–response analysis (IC_50_). Cells were treated with TMZ for 48h and IC_50_ was determined by MTT assay. **c** Colony formation assay. Cells were seeded in 6-well plates at a density of 1000 cells per well and cultured with fresh media containing TMZ (0, 50, 100µM as indicated) every other day for 10 days. Cells were fixed and stained with 4% formaldehyde, 0.05% crystal violet in PBS for 30min. Absorbance at 590nm was measured after dissolving with 10% acetic acid. The data obtained were normalized to the non-colony formation of non-treated parental controls. Means ± SD; *n* = 3; Student’s two-tailed *t*-test, *p < 0.05, **p < 0.01, ***p < 0.001.

To investigate the role of DAB2IP in TMZ-resistance, DAB2IP expression was modulated; endogenous DAB2IP was knocked down in the DAB2IP-high A172 cell line, and an exogenous DAB2IP plasmid was transfected into the DAB2IP-low LN229 cell line (Fig. 2a). Then, TMZ sensitivity was tested in these stable sub-cell lines (A172 control and knockdown cells, Con vs. KD; LN229 control and overexpression cells, Vc vs. OE). The results indicated that the knockdown of DAB2IP in A172 cells significantly increased resistance to TMZ (IC_50_ = 169.1 ± 16.7 µM compared to 71.6 ± 6.9 µM in the control), whereas the overexpression of DAB2IP in LN229 cells reduced TMZ-resistance (IC_50_ = 102.7 ± 19.3 µM compared to 224.6 ± 20.3 µM in Vc) (Fig. 2b). Consistent with TMZ sensitivity, the cell cycle analysis showed that the TMZ-sensitive A172 parental cells showed an accumulation of G2/M phase arrest after TMZ treatment, whereas the knockdown of DAB2IP decreased G2/M arrest (Supplementary Fig. S1).

In addition, the depletion of DAB2IP significantly promoted the inhibitory effect of TMZ on cell proliferation and colony formation (Fig. 2c). Since DAB2IP expression levels were not significantly affected by TMZ treatment (Supplementary Fig. S2), the manipulation of DAB2IP levels seemed to regulate TMZ sensitivity directly. Together, these results indicate that DAB2IP expression may be inversely correlated with TMZ-resistance in GBM cell lines.

### DAB2IP inhibits TMZ-induced autophagy

Numerous in vitro and in vivo studies have demonstrated that TMZ treatment induced apoptosis and autophagy simultaneously in GBM, and the pro-survival role of autophagy is considered part of the TMZ-resistance mechanisms^9,17–20^. When treated with TMZ, the protein expression levels of ATG5 and LC3B, representative autophagy markers, were increased only in TMZ-resistant LN18 and LN229 cells but not in TMZ-sensitive A172 and U373 cells (Supplementary Fig. S3a). To measure the level of autophagy, we analyzed the formation of acidic vesicular organelles (AVOs) which accumulate in acidic compartments such as autolysosomes. Consistently, AVOs were increased in both LN18 and LN229 cells (Supplementary Fig. S3b).

**Fig. 3:**
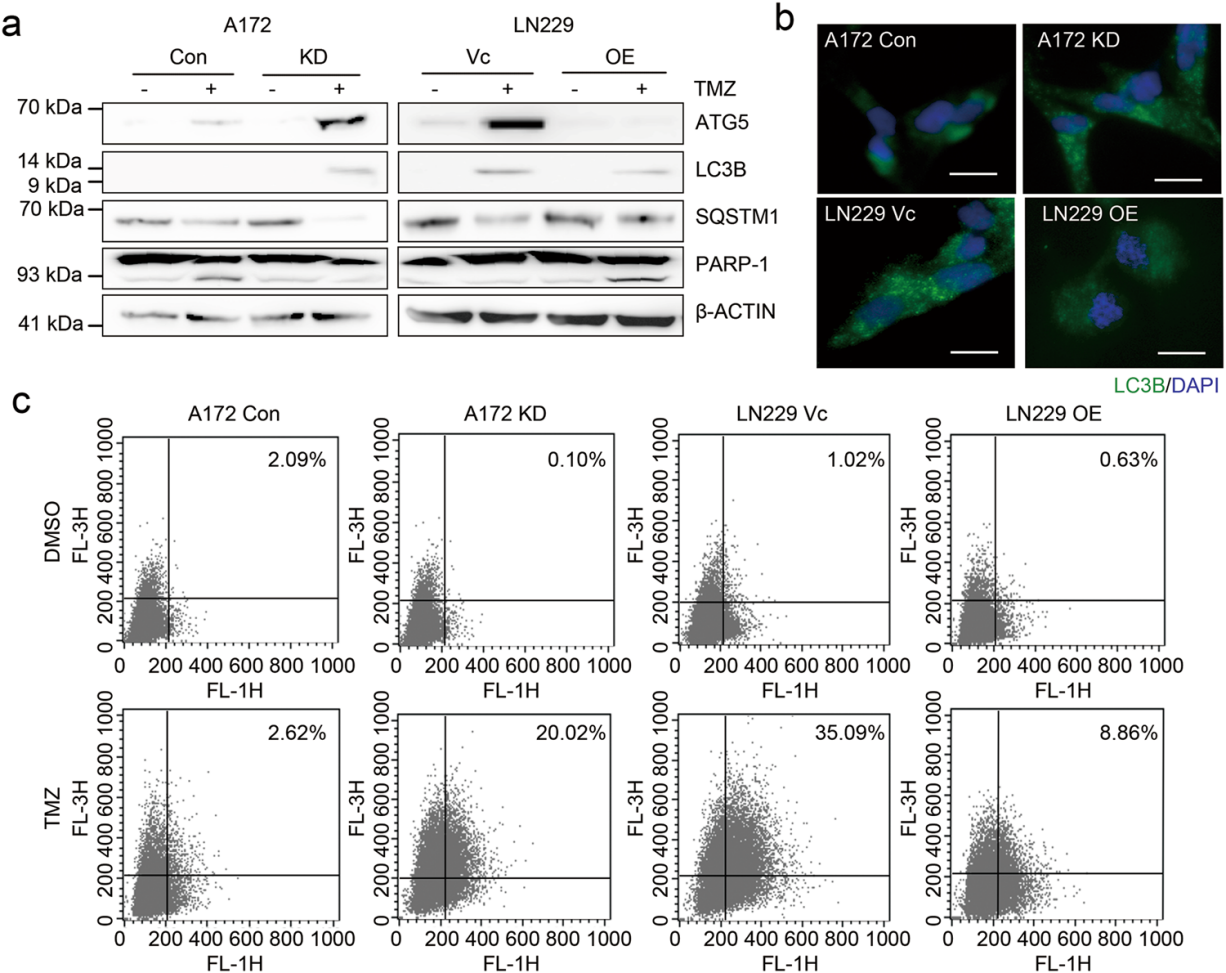
DAB2IP inhibits TMZ-induced autophagy. **a** Representative images of western blot against autophagy-related markers (ATG5, LC3B, SQSTM1, PARP-1) and β-actin. DAB2IP knock-downed A172 cells (KD), DAB2IP overexpressed LN229 cells (OE), and parental control cells were treated with 50µM TMZ for 48h followed by western blot analysis. **b** Representative immunofluorescence images against LC3. Cells were treated with 50µM TMZ for 48h followed by immunostaining. Scale bar, 20µm. **c** Flow-cytometry analysis for the detection of acidic vesicular organisms.

To investigate the functional impact of DAB2IP-loss on TMZ-induced autophagy, we analyzed the level of autophagy in DAB2IP-modulated cell lines upon TMZ treatment. Basal levels of ATG5 and LC3B were not detectable in either the TMZ-sensitive DAB2IP-high or TMZ-resistant DAB2IP-low cells. However, an increase of ATG5 and LC3B expression was observed in DAB2IP-low cells including A172 KD and LN229 Vc. noticeably, the autophagy substrate SQSTM1 was decreased in DAB2IP-low cells after TMZ treatment (Fig. 3a). In contrast, DAB2IP-high cells showed an increase of PARP-1 cleavage after treatment instead of an increase of ATG5 and LC3B (Fig. 3a). Moreover, the flow-cytometry analysis showed increased numbers of autophagosomes and autolysosomes in DAB2IP-low cells compared with those in DAB2IP-high cells (Fig. 3b, c). Collectively, our data suggest that DAB2IP-loss enhances autophagy activity leading to TMZ-resistance.

### DAB2IP regulates ATG9B expression in GBM cell lines

**Fig. 4:**
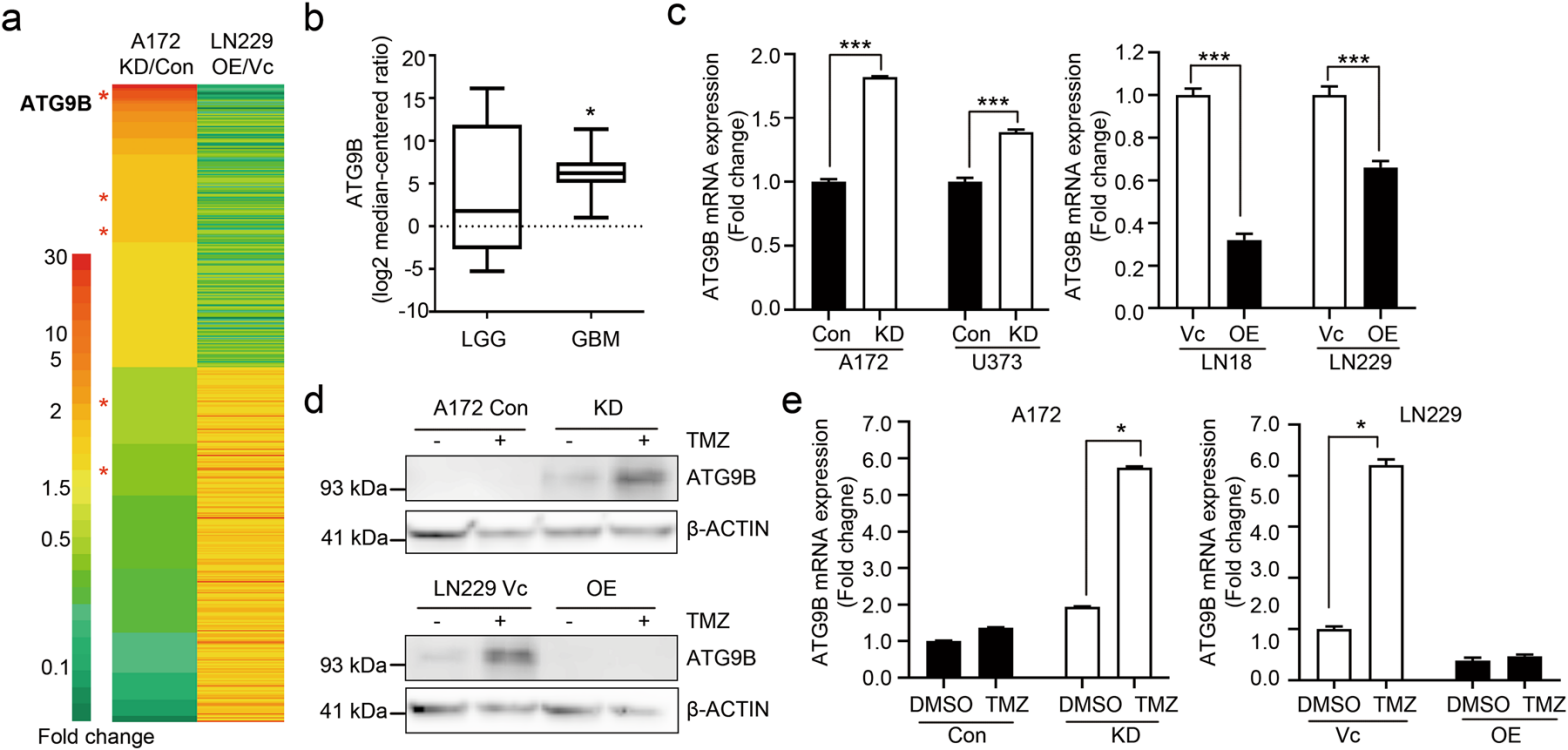
DAB2IP inhibits ATG9B expression. **a** Heatmaps showing differentially expressed genes detected by RNA sequencing (HiSeq 2000 platform, Illumina). Gene expression data were normalized to each of the parental control cell lines (Con or Vc). Red asterisks (*) indicate autophagy-related genes. **b** The levels of ATG9B expression in low-grade glioma (LGG) versus glioblastoma (GBM) tissues from TCGA. The horizontal lines in the box plots indicate the median, boundaries of the box indicate the 25th and 75th percentile, whiskers include 95% of the data. Unpaired *t*-test, *p < 0.05. **c** ATG9B mRNA expression detected by real-time PCR. **d**, **e** Representative images of western blot against ATG9B and β-actin. Cells were treated with 50µM TMZ for 48h as indicated. Black and white bars indicate DAB2IP-high and DAB2IP-low cell lines, respectively. Means ± SD; *n* = 3; Student’s two-tailed *t*-test, *p < 0.05, ***p < 0.001.

DAB2IP is known to regulate various biological processes including autophagy^21–23^. To reveal the molecular mechanism underlying DAB2IP regulation in TMZ-resistance, we performed RNA-Seq expression profiling in DAB2IP-high and -low cells. We identified 521 genes whose expression was significantly altered in both A172 and LN229 depends on DAB2IP expression levels. These included several genes in the autophagy pathway. Among these, *ATG9B* expression was the most significantly increased by DAB2IP knockdown in DAB2IP-high A172 cells, whereas it was decreased by DAB2IP overexpression in DAB2IP-low LN229 cells (Fig. 4a).

Autophagy-related (ATG) proteins have a key role in the formation of autophagosomes, the hallmark of autophagy^24,25^. ATG9B has been shown to be involved in the early phase of pre-autophagosomal structures (PAS) based on its accumulation in the PAS, however, the detailed function of *ATG9B* remains largely unknown^26^. Besides, unlike all other ubiquitous *ATG* genes, *ATG9B* shows tissue-specific expression patterns; *ATG9B* is abundant in the placenta and ovary but minimally expressed in other organs, including the brain^27^. However, through TCGA database analysis, we found significant increases of *ATG9B* in GBM tissues when compared to low-grade glioma (grade II), suggesting the clinical relevance of *ATG9B* in GBM (Fig. 4b).

We further confirmed an inverse expression pattern of DAB2IP and ATG9B by real-time PCR, as well as western blots (Fig. 4c, d). Furthermore, TMZ treatment induced further increases of ATG9B expression in DAB2IP-low cells suggesting the possible role of DAB2IP as a negative regulator of TMZ-induced autophagy (Fig. 4d, e).

### ATG9B enrichment is involved in TMZ-induced autophagy

**Fig. 5:**
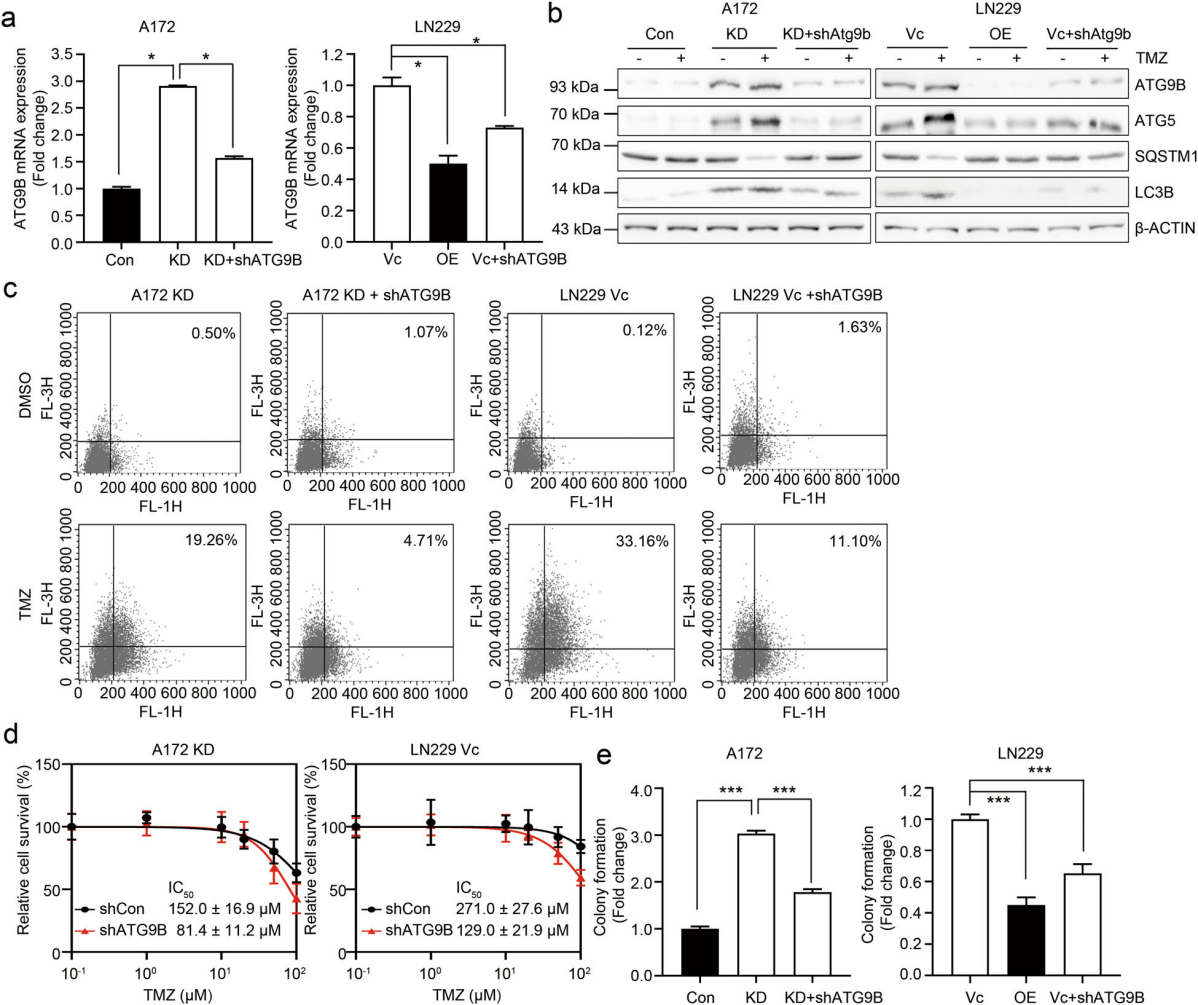
ATG9B enrichment is involved in TMZ-induced autophagy. **a** The mRNA expression of ATG9B analyzed by real-time PCR. pLKO-ATG9B plasmid (shATG9B) was transfected to DAB2IP-low A172 KD or LN229 Vc cells to knockdown ATG9B expression. Black and white bars indicate DAB2IP-high and DAB2IP-low cell lines, respectively. **b** Representative images of western blot against autophagy-related markers (ATG9B, ATG5, SQSTM1, LC3B) and β-actin. **c** Flow-cytometry analysis for the detection of acidic vesicular organisms. Flow data are the representative image of two biological experiments. **d** TMZ log–dose–response analysis (IC_50_). Cell viability was assessed by MTT assay. IC_50_ was calculated from curves constructed by plotting the cell survival versus TMZ concentration. The values are expressed as the mean of five technical replicates ± SD. **e** Colony formation assay. ATG9B knockdown cells and their control cells were seeded in 6-well plates at a density of 1000 cells per well. After 10 days of culture, cells were fixed and stained with 4% formaldehyde, 0.05% crystal violet in PBS for 30min. Absorbance at 590nm was measured after dissolving with 10% acetic acid. Black and white bars indicate DAB2IP-high and DAB2IP-low cell lines, respectively. Means ± SD; *n* = 3 in **a**, *n* = 6 in **e**; Student’s two-tailed *t*-test, *p < 0.05, ***p < 0.001.

To determine a direct role for ATG9B in the induction of autophagy in GBM, we next tested whether the level of autophagy changed by the downregulation of ATG9B in A172 KD and LN229 Vc cells (Fig. 5a). After treatment with TMZ, the knockdown of ATG9B expression resulted in significant decreases of ATG5 and LC3B, hallmarks of autophagy, which were increased in DAB2IP-low cells (Fig. 5b). As ATG9B is involved in the early step of autophagy, inhibition of ATG9B might lead to failure of the autophagy process^26^. To confirm the role of ATG9B on autolysosome formation, we measured AVOs. As shown in Fig. 5c, while DAB2IP-low cells displayed enriched AVOs after TMZ treatment (19.26% in A172 KD; 33.16% in LN229 Vc), the knockdown of ATG9B significantly blocked the formation of AVOs supporting the observation. Depending upon the cellular context, autophagy can lead to either abnormal cell growth or cell death^28^. In order to determine whether the inhibition of ATG9B was directly or indirectly linked to autophagy-mediated TMZ-resistance in GBM, we utilized both a cytotoxicity assay and a clonal growth assay. As predicted, the knockdown of ATG9B showed higher sensitivity to TMZ compared to the parental cells (Fig. 5d) and decreased the clonal growth in GMB cell lines as well (Fig. 5e). Together, these results strongly indicated the critical role of ATG9B in TMZ-induced autophagy and chemo-resistance regulated by DAB2IP.

### Wnt/β-catenin signaling pathway mediates the regulation of ATG9B by DAB2IP

**Fig. 6:**
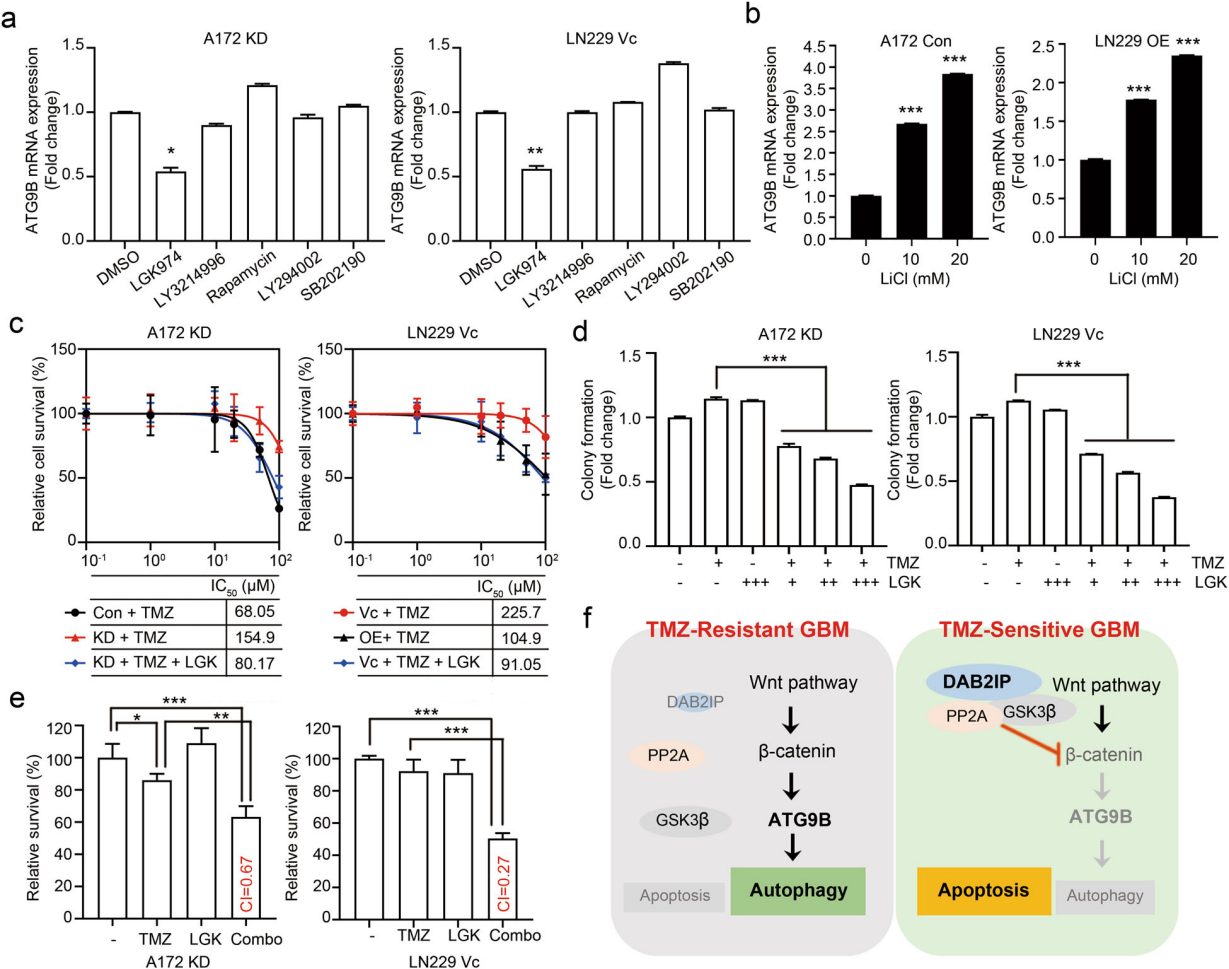
ATG9B expression was regulated through Wnt/β-catenin signaling pathway. **a**, **b** The mRNA expression of ATG9B detected by real-time PCR. DAB2IP-low cells were co-treated with 50µM TMZ and small molecule inhibitors (LGK974, 500nM; LY3214996, 50nM; Rapamycin, 10nM; LY294002 1µM; and SB202190, 10µM) for 48h before the analysis (**a**). DAB2IP-high cells were co-treated with 50µM TMZ and Wnt signaling activator LiCl for 48h before the analysis (**b**). **c** TMZ log–dose–response analysis (IC_50_). Cells were seeded in a 96-well and treated with increment doses of TMZ as indicated for 48h. LGK974 (200nM) was treated in combination with TMZ as indicated. **d** Colony formation assay. DAB2IP-low A172 KD and LN229 Vc cells were seeded in 6-well plates at a density of 1000 cells per well. TMZ, LGK974, and a combination of TMZ and LGK974 were treated to cells for 10 days. **e** Cell viability tested by MTT assay. Cells were treated with 50µM TMZ, 200nM LGK974, or a combination for 48h. Percent cell survival was normalized to untreated conditions. Drug synergistic effects were determined based on the combination index (CI). CI < 1, synergistic; CI = 1, additive; CI > 1, antagonistic effect. **f** The mechanism of DAB2IP involving in TMZ-resistance was illustrated. Means ± SD (*n* = 3 in **a**, **b**, **d**; *n* = 5 in **c**; *n* = 6 in **e**); Student’s two-tailed *t*-test, *p < 0.05, **p < 0.01, ***p < 0.001.

In addition to the function as a Ras-GAP, DAB2IP is known to regulate multiple signaling pathways, including the PI3K/Akt, Ask1/JNK, and Wnt/β-catenin pathways^29^. To further elucidate the underlying mechanism of DAB2IP in regulating TMZ-induced autophagy, we explored the signaling pathways involved in the suppression of ATG9B expression. DAB2IP-low A172 KD and LN229 Vc cells were treated with TMZ in combination with the inhibitors of specific signaling pathways. Optimal concentrations of the inhibitors that could effectively block each targeted-signaling pathway without altering cell survival were used (Supplementary Fig. S4). The results showed that only the Wnt/β-catenin pathway blockage among those tested suppressed ATG9B expression (Fig. 6a). Although the PI3K-mTOR pathway has been reported to negatively regulate autophagy in various cancers including GBM^30–32^, ATG9B mRNA expression was not affected by either rapamycin or LY294002 (Fig. 6a). To activate the Wnt/β-catenin signaling pathway in DAB2IP-high cells, the cells were treated with lithium chloride (LiCl) which can activate the Wnt signaling pathway by enhancing β-catenin stabilization. The results showed a LiCl dose-dependent increase of ATG9B expression suggesting that ATG9B expression was mainly regulated through the Wnt/β-catenin pathway (Fig. 6b). To further confirm whether Wnt/β-catenin blockage could affect TMZ chemo-sensitivity, the effect of the Wnt inhibitor LGK974 in combination with TMZ was tested. As shown in Fig. 6c, d, combination treatment with LGK974, and TMZ decreased the IC_50_ of TMZ in DAB2IP-low cells (Fig. 6c) and also inhibited colony formation (Fig. 6d). Furthermore, the results from combination index analysis showed that the combination regimen exhibited a synergistic effect (A172 KD, CI = 0.666 and LN229 Vc, CI = 0.271, respectively) in the cells suggesting the combination of a Wnt inhibitor with TMZ as a novel therapeutic strategy to avoid TMZ-induced autophagy (Fig. 6e). Taken together, these results demonstrate that ATG9B, a key downstream component of TMZ-induced autophagy, is regulated by the Wnt/β-catenin signaling pathway.

In conclusion, DAB2IP suppressed the Wnt/β-catenin signaling pathway involved in TMZ-induced autophagy as well as TMZ-resistance. Therefore, utilizing a Wnt inhibitor may synergize the effect of conventional TMZ therapy in TMZ-resistant GBM (Fig. 6f).

## Discussion

Although TMZ-based chemotherapy along with focal radiotherapy has been widely used as the standardized first-line therapeutic strategy in GBM therapy, TMZ-resistance eventually occurs in almost all patients, and the recurrent tumors are generally more aggressive and malignant^3,4^. Numerous investigations have focused on MGMT as the major contributor to TMZ-resistance^4,33^. Clinical studies have shown that elevated MGMT protein levels or the lack of MGMT promoter methylation were associated with TMZ-resistance in GBM tumors^34,35^. However, some MGMT-deficient GBMs are resistant to TMZ, and the underlying molecular mechanisms remain unclear^33,36^. Understanding the molecular mechanisms of the chemo-resistance of GBM is pivotal to developing new therapeutic strategies to achieve ultimate curative therapies. Here, we have demonstrated a novel mechanism of the DAB2IP/Wnt/β-catenin/ATG9B axis in the regulation of TMZ-resistance, which suggests DAB2IP and ATG9B as new components of the TMZ-resistance mechanism and may provide a potential therapeutic strategy.

Autophagy is an evolutionarily conserved cellular catabolic process that has a critical role in maintaining cellular homeostasis through the lysosomal degradation of unnecessary or dysfunctional components during metabolic stress, pathogenic infection, radiation damage, and drug therapy^37^. Since it has been served in both pro-survival and pro-apoptotic mechanisms, the role of autophagy in cancer cell biology is complicated and evolves throughout tumorigenesis^28,30,38,39^. In this study, TMZ-resistant GBM cell lines showed enhanced autophagic processes, such as increased expression of the autophagy-related gene as well as acidic autolysosome formation after TMZ treatment. Besides, the loss of DAB2IP in GBM cell lines increased TMZ-resistance. The loss of DAB2IP has been confirmed to be associated with poor prognosis in many cancers^7,10,21^, however, its role in TMZ-resistance in GBM is largely undefined. Here, we report that the loss of DAB2IP enhances autophagic flux after the treatment of TMZ in GBM cell lines. Mechanistically, we have identified that autophagy-related ATG9B expression is increased by the loss of DAB2IP. ATG proteins are known to be required for the formation of autophagosomes, the initial vacuoles that sequester cytoplasmic material before its delivery to lysosomes^24,25^. The detailed function of ATG9B remains unclear yet, however, several studies have suggested that ATG9B might be involved in the early phase of PAS formation since many ATG proteins are not localized in the PAS in budding yeast lacking Atg9^40,41^. In this study, the knockdown of ATG9B in DAB2IP-low GBM cells fail to induce autophagy after TMZ treatment suggesting ATG9B as a critical player during the autophagy process.

In addition to key downstream players, we also have identified a signaling pathway involved in TMZ-induced autophagy mediating TMZ-resistance. Over the past decades, the mTOR signaling pathway has been considered as a central regulator of autophagy by modulating multiple aspects of the autophagy process from initiation to termination^42–44^. In addition to this, several studies have reported the complex interplay between the Wnt/β-catenin pathway and autophagy in several types of cancers^45–49^. Our results show that Wnt/β-catenin activation induces the upregulation of ATG9B expression leading to autophagy, whereas Wnt/β-catenin blockage inhibits ATG9B expression as well as TMZ-resistance suggesting a link between Wnt/β-catenin signaling and TMZ-induced autophagy. DAB2IP is known to function as a key scaffold protein to recruit PP2A to activate GSK3β to inhibit Wnt/β-catenin signaling^21^. In this study, we show that blocking the Wnt/β-catenin signaling pathway using a small molecule inhibitor significantly decrease TMZ-induced ATG9B expression as well as TMZ-induced autophagy. Conversely, activation of the Wnt/β-catenin signaling pathway using LiCl in TMZ-sensitive GBM cell lines increases ATG9B expression after TMZ treatment suggesting that the Wnt/β-catenin pathway is involved in regulating TMZ-induced autophagy in GBM.

The molecular mechanism underlying TMZ-induced autophagy demonstrated in this study may provide a new rationale in anti-GBM therapy using TMZ in combination with a clinically available small-molecule inhibitor of the Wnt/β-catenin pathway.

## Materials and methods

### Cell culture

Human glioblastoma cell lines LN18 and LN229 cells were recently purchased from ATCC and cultured in Hyclone^TM^ Dulbecco’s Modified Eagles Medium (DMEM, ThermoFisher Scientific) containing a final concentration of 5% fetal bovine serum (FBS) and antibiotics (penicillin/streptomycin 100 IU/ml, Gibco) at 37 °C in a 5% CO_2_ atmosphere. Human glioblastoma cell lines A172 and U373 were kindly provided by Dr. Kyu Lim, College of Medicine, Chungnam National University, Korea^50^. These cell lines were cultured in DMEM containing 10% FBS and antibiotics at 37 °C in a 5% CO_2_ atmosphere. All cell lines were mycoplasma free and routinely tested by PCR amplification.

### Plasmids construction and reagents

The DAB2IP expression plasmid was prepared as described previously^9,21^, and stable clones were selected by G418 at a concentration of 1000 µg/ml after 3–4 weeks. pGIPZ-DAB2IP-lentiviral-shRNAmir and pGIPZ-non-silencing-lentiviral-shRNAmir were purchased from Open biosystems, and infection was followed according to the manufacturer’s protocol. After infection, cells were selected by puromycin at a concentration of 0.2 μg/ml for 3–4 weeks. Primary antibodies used were as follow: rabbit polyclonal anti-DAB2IP (ab87811), anti-ATG9B (ab108107), and anti-ATG5 (ab228668) were purchased from Abcam, rabbit monoclonal anti-actin (Cat#4970), anti-LC3b (Cat#12741), anti-phospho-β-catenin (Cat#9565), anti-phospho-GSK3β (Cat#5558), anti-phospho-Erk1/2 (Cat#4370S), anti-total-Erk1/2 (Cat#9107S), anti-phospho-p70S6K (Cat#9205), anti-p70S6K (Cat#9202), anti-phospho-Akt (Cat#4060), anti-Akt (Cat#4691), anti-phospho-p38 (Cat#4511), anti-p38 (Cat#9212), and anti-PARP-1 (Cat#9532) were purchased from Cell Signaling Technology. Anti-SQSTM1 was purchased from an R&D system (Cat#MAB8028R).

Temozolomide (Cat#14153, TMZ) was purchased from Cayman chemical company, LGK974 (S7143), LY3214996 (S8534), LY294002 (S1105), SB202190 (S1077), and Rapamycin (S1039) were purchased from Selleckchem.

### In vitro cytotoxicity assay

Cells (5 × 10^3^) were plated in 96-well plates. After attachment, cells were treated with different doses of TMZ for 72 h. MTT (3-(4,5-dimethyl-2-thiazolyl)-2,5-diphenyl-2H-tetrazolium bromide) dye solution was added to each well and incubated for 4 h at 37 °C, followed by the addition of DMSO to lyse the cells and solubilize the colored crystals. Cell viability was assessed by measuring the absorbance at 570 nm in a spectrophotometer. IC_50_ was determined with cytotoxicity results which were more than 50% at screening concentrations, and calculated using Fit spline/LOWWESS analysis interpolating unknowns from a standard curve.

Drug synergistic effects were determined based on combination index (CI)^51^. The CI was calculated using the formula: ${\mathrm{CI = }}\frac{{{\mathrm{CA}},{\mathrm{x}}}}{{{\mathrm{ICx}},{\mathrm{A}}}} + \frac{{{\mathrm{CB}},{\mathrm{x}}}}{{{\mathrm{ICx}},{\mathrm{B}}}}$, where C_A,X_ and C_B,X_ are the concentration of drugs A and B used in combination to achieve X% drug effect. IC_X,A_ and IC_X,B_ are the concentrations for single agents to achieve the same effect. CI < 1, synergistic; CI = 1, additive; CI > 1, antagonistic effect.

### RNA isolation and quantitative real-time PCR

Total RNA was extracted with the ReliaPrep RNA Miniprep System (Promega) and 1 μg RNA was reverse transcribed with the Luna Script (Promega). Real-time PCR analysis was performed by using MIC real-time PCR system (MIC) and PowerUP SYBR Premix (Applied bioscience). The relative level of target mRNA was evaluated using the Ct method, and the fold change was determined by calculating 2^−∆∆Ct^ normalized with 18S rRNA. All experiments were repeated at least twice to triplicate results.

### Western blot analysis

For western blot analysis, cells were lysed using RIPA buffer containing both protease inhibitor and phosphatase inhibitor and subjected to electrophoresis on 15% SDS-polyacrylamide gel. Separated proteins were transferred to PVDF membranes (Millipore) and blocked with 5% skimmed milk (w/v) for 1 h. Membranes were then incubated with primary antibody overnight, and antibody binding was detected using the appropriate secondary antibody coupled with horseradish peroxidase. Signals were visualized using LAS4000 (GE Lifesciences) after adding Clarity ECL Western Substrate (BioRad).

### Colony assay

Cells were plated in 6-well plates at the clonal density of 1000 cells per well and treated with incremental doses of TMZ. After 10 days of culture, colonies were fixed and stained with 4% formaldehyde in PBS containing 0.02% crystal violet for 30 min, and washed with tap water. Absorbance at 590 nm was measured after solved the crystal violet with 10% acetic acid.

### Quantification of acidic vesicular organelles (Acridine orange staining)

Acridine orange (A8065, Sigma) was used to measure the number of acidic vesicular organelles (AVOs) in cells. After TMZ treatment, cells were incubated with Acridine orange (1 μg/ml) for 15 min at 37 °C. Cells were then washed twice with PBS followed by flow-cytometry analysis (FACS Calibur, BD Biosciences).

### RNA sequencing and analysis

The samples were prepared for sequencing using the ReliaPrep RNA Miniprep System (Promega) according to the manufacturer’s instructions and sequenced on a HiSeq 2000 platform (Illumina). The 101-bp sequenced paired-end reads were mapped to the hg19 reference human genome using the STAR 2-pass methods^52^. HTSeq was used to count the reads aligned to each gene based on the Ensemble gene set^53^. The normalized read counts were applied to principal component analysis or clustering analysis, which was conducted through R and Cluster 3.0 and visualized via Java Treeview^53–55^.

### Statistical analysis

The data were expressed as the mean ± SD. Statistical analysis was performed using GraphPad Prism 8 software. Student’s two-tailed *t*-test was used for the determination of statistical relevance between groups, and *p* < 0.05 was considered statistically significant.

## Supplementary information

Supplemental Figure S1

Supplemental Figure S2

Supplemental Figure S3

Supplemental Figure S4

Supplemental Figure legends
