# Supplementary material for: Wnt/β-catenin signaling pathway induces autophagy-mediated temozolomide-resistance in human glioblastoma
Source: Cell Death Dis. 2020 Sep 17;11(9):771. doi: 10.1038/s41419-020-02988-8 (PMC7498596; doi:10.1038/s41419-020-02988-8)
Supplement: Supplementary file 5 — Supplemental Figure legends [file 41419_2020_2988_MOESM5_ESM.docx]

**Supplementary Information and Figure legends**

**Figure S1. DAB2IP increases G2/M phase arrest after TMZ treatment**

Cell cycle analysis in DAB2IP-high and DAB2IP-low cells after TMZ treatment. A172 Con and KD cells were treated with 50 µM TMZ for indicated times. Propidium iodide (PI) staining was used to detect the distribution of cells.

**Figure S2. DAB2IP expression was not affected by TMZ treatment**

The mRNA expression of DAB2IP analyzed by real-time PCR. Cells were treated with TMZ for 48 hours before analysis. Black and white bars indicate DAB2IP-high and DAB2IP-low cell lines, respectively. means ± SD; n=3; Student’s two tailed t-test, **p<0.01, ***<p<0.001.

**Figure S3. TMZ-resistant cells increase autophagy after TMZ treatment**

(a) Representative images of Western blot against autophagy-related markers (ATG9B, ATG5, SQSTM1, LC3B) and β-actin. Four glioblastoma cell lines were treated with 50 µM TMZ for 48 hours. (b) Flow cytometry analysis for the detection of acidic vesicular organisms.

**Figure S4. The effect of individual small molecule inhibitor on GBM cell survival**

(a) Representative images of Western blot against downstream mediator of each signaling pathway (phospho-β-catenin, phospho-Erk1/2, phospho-p70S6k, phospho-Akt, and phospho-p38 ). (b) Cell viability tested by MTT assay. DAB2IP-low cells were treated with LGK974 (500 nM), LY3214996 (50 nM), Rapamycin (10 nM), LY294002 (1 µM), and SB202190 (10 µM) for 48 hours. Means ± SD; n=5; Student’s two tailed t-test.
